# Exploring the feasibility and acceptability of a school‐based self‐referral intervention for emotional difficulties in older adolescents: qualitative perspectives from students and school staff

**DOI:** 10.1111/camh.12234

**Published:** 2017-08-24

**Authors:** Lynn McKeague, Nicola Morant, Emily Blackshaw, June S. L. Brown

**Affiliations:** ^1^ Department of Psychology University of Winchester Winchester UK; ^2^ Division of Psychiatry University College London London UK; ^3^ Department of Psychology Institute of Psychiatry, Psychology & Neuroscience King's College London London UK

**Keywords:** Adolescence, depression, anxiety, school, qualitative methods

## Abstract

**Background:**

Adolescents with emotional difficulties need accessible, acceptable and evidence‐based mental health interventions. Self‐referral workshops (DISCOVER workshops) were offered to stressed 16‐ to 19‐year olds in 10 Inner London schools.

**Method:**

Semistructured interviews were conducted with three groups of participants: students who attended a 1‐day workshop (*n* = 15); students who initially showed interest in the DISCOVER workshop programme, but decided not to take part (*n *=* *9); and school staff who helped organise the programme in their schools (*n *=* *10). Students were purposively sampled to ensure that those from Black and minority ethnic (BME) backgrounds were represented. Data were analysed using thematic analysis.

**Results:**

The accounts generally indicate that the delivery and evaluation of this intervention is perceived as feasible and acceptable. Students, including those from BME backgrounds, described the setting as suitable and reported that the workshop helped them develop new understandings of stress and how to handle it. They expressed a preference for engaging and interactive activities, and valued a personalised approach to workshop provision. School staff felt that the workshop was in line with school values. They described some logistical barriers to providing the workshops in school settings, and expressed a desire for more information about the workshop in order to provide follow‐up support. The main reason students gave for nonparticipation was limited time.

**Conclusions:**

Findings are discussed in relation to increasing the feasibility of implementing school‐based psychological interventions and the value of providing access to mental health support in schools.

**Key Practitioner Message**

- The DISCOVER ‘How to Handle Stress’ workshop programme is acceptable to both students and school staff.
- Results suggest that school‐based group CBT interventions for 16‐ to 19‐year olds should be engaging, interactive and personalised.
- Providing psychological support in the schools makes it more accessible for hard‐to‐reach groups, such as BME students.
- Collaboration and clear communication between psychologists and school staff, and working closely with administrative staff to avoid logistical issues are key to workshop delivery in the school setting.

## Introduction

It is estimated that half of all lifetime mental health problems have their onset by midadolescence (Kessler et al., 2005). Mood and anxiety disorders are known to be prevalent at this time (Costello, Mustillo, Erkanli, Keeler, & Angold, 2003) and are associated with a range of adverse outcomes, including increased suicide risk (Gould, Greenberg, Velting, & Shaffer, 2003). Given reluctance to seek help from specialist services (Rickwood, Deane, Wilson, & Ciarrochi, 2005), there is great potential to provide mental health support in the school setting (Calear & Christensen, 2010; Merry et al., 2012). Although efforts to develop school‐based interventions for depression and anxiety have increased in recent years, studies investigating their effectiveness have yielded mixed results (Stallard, 2013). Existing interventions have primarily been evaluated by measuring quantitative psychological outcomes (Corrieri et al., 2013; Spence & Shortt, 2007), with some researchers also collecting data on participant satisfaction to supplement data on intervention outcomes (e.g. Garmy, Berg, & Clausson, 2015; Taylor et al., 2014).

Qualitative methods are of particular value in exploring the experiences of those receiving and delivering services, as they allow for the influence of sociocultural and contextual factors. This may help elucidate the reasons for discrepant trial results, or the difficulties of implementation or sustainability of complex interventions (Campbell et al., 2000). Qualitative approaches have allowed in‐depth explorations of young people's perceptions of mental health services, for example impressions of professionals who deliver them (Day, Carey, & Surgenor, 2006; Donnellan, Murray, & Harrison, 2012). They have also allowed better understanding of factors that promote or undermine effective delivery of classroom‐based Cognitive Behaviour Therapy (CBT) interventions (Taylor et al., 2014). Finally, use of qualitative approaches gives students and school staff opportunities to recommend changes for further intervention development (Boyle, Lynch, Lyon, & Williams, 2011).

The DISCOVER ‘How to Handle Stress’ workshop programme is a self‐referral school‐based group intervention designed for stressed students in sixth form (16–19 years). The programme aims to provide early intervention for adolescents with emotional difficulties, applying cognitive‐behavioural strategies within a broad stress‐coping paradigm. The programme does not specifically screen for anxiety or depression; participants self‐refer owing to self‐perceived need for psychological support in managing common adolescent stressors. The self‐referral system was based on the work of Brown, Boardman, Whittinger, and Ashworth (2010) which showed that people referring themselves often had high levels of symptoms and were more representative of the population, in terms of ethnicity, than GP referrals. The programme comprises a 1‐day workshop, for up to 15 students, codelivered by two clinical psychologists and one assistant psychologist, with a supporting workbook and personalised telephone follow‐up. The workshop was adapted from self‐referral psycho‐educational CBT workshops originally developed for adults in the community (Brown, Cochrane, & Hancox, 2000). The version for teenagers was adapted (Michelson et al., 2016; Sclare, Michelson, Malpass, Coster, & Brown, 2015) to focus on the stresses of older adolescence. In the workshop, CBT techniques are outlined and practised, using presentations, videos, role‐play and a goal‐setting task. The DISCOVER workshop is designed to normalise common emotional difficulties and to provide accessible support by addressing barriers that typically deter adolescents from presenting at services, such as concerns about stigmatisation (Sclare et al., 2015). Pilot study results were promising, with high levels of uptake among “hard‐to‐reach” groups, such as Black and minority ethnic (BME) students and those with no previous experience of formal mental health help‐seeking (Sclare et al., 2015). The suitability of the workshop programme for these groups is important, given that these young people may be more likely to experience barriers (e.g. stigma) to seeking professional help, compared to their peers (Bradby et al., 2007; Lamb, Bower, Rogers, Dowrick, & Gask, 2012).

The current study is the qualitative component of a cluster randomised controlled trial (RCT) investigating the feasibility of the DISCOVER workshop programme for students in UK sixth forms (aged 16–19) in 10 Inner London schools, described in Brown et al. (2017). Schools were randomised into experimental and control groups.

## Methods

### Aims

This qualitative study aimed to investigate the feasibility and acceptability of the DISCOVER workshop programme. Descriptions of the methods and findings below follow the COREQ checklist (Tong, Sainsbury, & Craig, 2007).

### Participants and setting

The RCT was conducted in Inner London state secondary schools (nine mixed, one single sex). Eligibility criteria for the DISCOVER trial were that students were in Year 12 or 13, that they were over 16 years old, had fluent English, wished to receive psychological help for emotional difficulties, were willing and able to attend a 1‐day psychological workshop on school premises, and were able to provide informed written consent to participate (as described in Michelson et al., 2016). The areas in which the research was conducted are considered economically deprived and ethnically diverse (Lambeth Council, 2014; London Borough of Southwark, 2013). The referral pathway was self‐referral; those who took part in the intervention self‐selected. School staff did not attend the workshop, nor did they inform intervention content. They were however asked to approach vulnerable individuals and encourage them to attend and to organise logistical aspects of running the programme within the school. Students were eligible to participate regardless of whether they had ever been diagnosed with depression/anxiety and were not screened before taking part.

### Participants

(1) Workshop attenders, (2) students who showed interest in DISCOVER workshops but did not participate, and (3) school staff who helped organise the DISCOVER workshop in their school.

### Sampling and recruitment

Participants were primarily recruited via telephone, having provided their contact details to the DISCOVER team in the initial stages of recruitment for the workshop programme. Participants were interviewed on one occasion only during school term‐time.

#### (1) Workshop attenders

Purposive sampling was used to obtain a sample of respondents who reflected the overall group of workshop attenders in terms of gender, ethnicity and past experience of seeking mental health support. In total, 21 students from the experimental group schools were contacted, until the sample size reached 15 participants. Non‐White students were oversampled as we had a particular interest in exploring intervention acceptability for this group. Interviews with workshop attenders were conducted approximately 4 months after the DISCOVER workshop, after the RCT follow‐up.

#### (2) Nonparticipants

Nonparticipants were recruited (*n *=* *9) on a rolling basis as soon as possible after they decided they did not want to take part, with 26 students contacted in total. Efforts were made to contact and recruit at least one participant from each experimental and control group school, but this did not prove possible.

#### (3) School staff

Staff were recruited from experimental group schools. Fifteen individuals were invited to be interviewed and 10 agreed. Interviews were conducted at a time suitable to participant availability approximately 5 months after the workshop, after the RCT follow‐up.

### Data collection

Semistructured interviews were conducted in participating schools by the first author, who had no involvement in delivery of the intervention, but was part of the DISCOVER research team. Interviews focused equally on positive and negative aspects of participants’ experiences, and explored the following topics, using primarily open questions, with some closed questions: the recruitment process; experiences of participating in the workshop; self‐perceived impact of the workshop; the feasibility of DISCOVER as a school‐based intervention; and the workshop's ability to meet young people's needs. Demographic information was also collected. For student interviews, a teenage advisory group, comprising eight teenagers not involved in the study, helped provide views on the appropriateness of topics and question wording. Interviews were conducted in a quiet room in the participant's school.

Interview schedules differed for the three participant groups. Workshop attenders were asked about their experiences of participating in the DISCOVER programme. Interviews with nonparticipants focused on reasons for deciding not to proceed with the intervention. School staff interviews explored the process of hosting the programme, and the impact of the programme on participating students. Interviews ranged in length from 6 min 52 s (nonparticipant) to 52 min 42 s (staff member). Brief field notes and interview summaries were compiled after each interview.

### Ethical considerations

Ethics approval for the DISCOVER Project was granted by the Health Research Authority NREC Committee London – Camberwell St Giles: ref 14/LO/1416. All participants provided written informed consent and consent for their interview to be audio‐recorded.

### Analysis

Data were transcribed verbatim and analysed in line with Braun and Clarke's approach to thematic analysis (Braun & Clarke, 2006, 2013). Analysis was primarily data‐driven, with a priori concerns to explore feasibility and acceptability of the intervention, and was not conducted from a particular theoretical standpoint. For each subsample, three researchers (LM, NM and EB) read a sample of interviews and met to discuss possible codes and themes. Coding frames were devised by the first author who used NVivo V.10 to code the data. The second and third authors reviewed the coding. Queries about coding decisions were discussed and the analysis continued through to the write‐up of study findings. Direct quotes from participant interviews are used to give voice to themes that were evident in the data. Both commonalities and variations within and between the subgroups were explored.

## Results

### Participants

The study included three subsamples, workshop attenders (*n *=* *15), nonparticipants (*n *=* *9) and staff (*n *=* *10). Workshop attenders and nonparticipants were predominantly from BME groups, female and had not previously sought support, with an average age of 17.59 years and 17.44 years respectively (see Table 1). Characteristics of these participants were reflective of the larger population of workshop attenders, with the majority (over 70% in both subsamples) from BME groups.

**Table 1:** Demographic details for study subsamples

	Workshop attenders *N* = 15	Nonparticipants *N* = 9	School staff *N* = 10
Mean age (years)	17.59	17.44	38.28
Age range (years)	16.58–19.33	16.42–18.33	28.00–55.58
Gender (female)	12 (80.0%)	5 (55.56%)	8 (80%)
*Ethnicity*			
Black British, African	6 (40%)	4 (44.44%)	0 (0%)
Black British, Caribbean	3 (20%)	0 (0%)	3 (30%)
White British	4 (26.67%)	2 (22.22%)	5 (50%)
Other BME group	2 (13.33%)	3 (33.33%)	2 (20%)
Previously received help for mental health (i.e. counsellor; school counsellor; GP; teacher; youth club counsellor; the DISCOVER programme; therapist)	5 (33.33%)	3 (33.33%)	–
*Position in school*			
Head/Director of Sixth Form	–	–	3 (30%)
Deputy Head of Sixth Form	–	–	2 (20%)
Sixth Form Administrator/Manager	–	–	2 (20%)
Student Support Officer	–	–	1 (10%)
Lead Teacher High Achievers	–	–	1 (10%)
Head of year 13	–	–	1 (10%)

School staff were also predominantly female and 50% of the sample identified as White British. The majority of school staff held senior school roles (such as heads of sixth form), while two held administrative positions.

### Presentation of results: Overview

Results are presented in two sections. The first examines students’ experiences of actually participating in the DISCOVER workshop, and the perceived benefits or problems in taking part. The second section focuses on the acceptability and feasibility of delivery of the DISCOVER workshop in schools, drawing on the perspectives of workshop attenders, nonparticipants and school staff.

### Section 1: Experiences of participating in the DISCOVER workshop and perceived impacts

#### Understanding and managing stress

All 15 students indicated that the workshop had helped them to understand their stress or made them aware of stress management techniques. In terms of academic outcomes, many (*n *=* *9) said that their time management or planning had improved since taking part in the workshop.

I think it's made me think more about where the stress came from and that there are ways to deal with it rather than just freaking out. [P005]

…DISCOVER helped me with considering different ways of handling stress… [P004]

I'm not as stressed as I used to be, em, and I don't, like find myself needing to be worried about anything as much. Except for exams obviously… [P014]

As part of the workshop programme, students were encouraged to set a personal goal and were offered follow‐up telephone reviews with a workshop leader to monitor goal progress. All White British students (*n *=* *4) who were interviewed described the process of setting a goal in positive terms or said that it was ‘easy’ to decide on a goal. In contrast, negative perceptions of goal setting were apparent among some of the BME students (*n *=* *4). One [005] described setting a goal as ‘worrying’, because of the anticipation that she might not achieve it. Another student [P008] found the goal‐setting task difficult because his goals were constantly changing: “*…with me I've gotta keep changing mine*.” Of the students who reported experiencing difficulty with the goal‐setting exercise, none had previous experience of seeking formal support for mental health issues.

A small number of students described experiencing difficulties in using the techniques following the workshop, for example, due to challenges posed by increasing academic pressure and impending exam season. Two students thought that the workshop may not have a lasting impact.

…in the beginning […] it was more helpful, because […] it would have been fresh in my mind. [P011]

When asked specifically, all 15 said that they would recommend the DISCOVER workshop to friends, and several suggested it would be suitable for those who were stressed or studying for exams. In sum, the workshops were perceived as helpful to recognising sources of stress and identifying ways to deal with it, even though difficulties sustaining changes were reported.

#### Preference for engaging and interactive content

Several students (*n *=* *8) described the workshops as engaging, interactive or ‘different’ (in terms of including new ideas or techniques). They liked the variety of techniques used, the use of PowerPoint presentations and the workshop booklet. They preferred the more active and interactive components of the workshop day, with all participants commenting that they liked the videos used and/or could relate to the video character(s).

… the ones [techniques] that the workshop delivered were quite different and quite unique so they sort of made it easier to deal with things because there's stuff that you haven't really done before. [P007]

It [the workshop day] was great, we did, it was a whole day, we did so many activities, we learnt so many things, we tried new things, it was really fun. [p015]

…there was loads of different activities, not just reading and listening and sitting down, so it was interactive. [P009]

Interviewees were asked to comment on specific techniques or topics included in the workshop that they found helpful or unhelpful. The techniques and topics most commonly described favourably were: sleep (*n *=* *10), time management (*n *=* *9), mindfulness (*n *=* *8) and relaxation (*n *=* *7). Although some students said they found certain techniques unhelpful, 14 out of the 16 techniques/topics were liked or considered helpful by at least one student and 10 were reported to have been used by at least one of the students following the workshop. There were no clear patterns across participants in techniques that were disliked or the reasons given for this, which included not finding them novel, not understanding them, finding them hard to use in practice or not enjoying them.

Participants generally liked receiving a follow‐up phone call from one of the psychologists, describing this primarily as a means for the psychologists to ‘check‐up on them’, and an opportunity to request additional support.

#### The importance of an individualised approach

Students valued a personalised approach to workshop provision, for example, when the psychologists asked them to describe their lived experience of stress. Over one third of the sample (*n *=* *6) were content with the level of interaction between themselves and the psychologists.

[the workshop was] really interactive and because there wasn't a really large group of people, there was about 12 of us, it was quite individual as well. So personally I feel like that I got, got quite a good amount of attention and my questions were answered in quite detail [sic] because we had the time to do it. [P007]

However, a further third (*n *=* *5) thought the workshop was not individualised enough or that there was not enough opportunity for one‐to‐one interaction with the psychologists. They thought that the interactive nature of the workshop could be strengthened by, for example, more one‐to‐one opportunities, and working in smaller groups.

…helping young people that are feeling stressed, the best thing to do would be talk to them about their individual circumstance if they're willing to tell you their personal lives, ‘cause if they do then you know, you sort of know what angle to talk to them from… [P012]

### Section 2: Acceptability and feasibility of delivery in the school setting

#### Section 2.1: Perspectives of workshop attenders

##### Attending a workshop in the school setting

Six students described the convenience of workshops being held at school, and a further six described the setting as familiar, comfortable, safe and/or secure.

…it was quite good doing it in school, ‘cause we're all comfortable with our surroundings […] whereas if we done it in a place we've never been to before, we'd be a bit, like, on edge. [P010]

A smaller number (*n *=* *3) described a conflict between attending the workshop and missing lesson time. They felt that, lasting a full school day, the workshop took up too much of their time and recommended ways of altering the timescale of the workshop, such as spreading its content over two half‐days.

I think it just took a lot of time. It took a whole school day and for me that's really a lot of information that I missed and had to catch up on. [P001]

Two students suggested that a different location might be beneficial, with one expressing the concern that privacy and confidentiality might not be fully assured in the school setting.

##### Experience of a group‐based workshop

Several students (*n *=* *6) said they benefitted from hearing peers sharing information about themselves which led to realising that other people shared similar experiences and increased reassurance and reduced feelings of isolation. Some students (*n *=* *4) described feeling more comfortable about sharing personal information as the day progressed. A small number (*n *=* *3) commented that the size of the group was important in determining how willing they were to make these disclosures.

It was nice to see what other people thought and how they dealt with stress and what they felt stress was like. [P001]

… since it was a small group, we wouldn't feel intimidated to just tell people stuff. It was more confidential in a sense. [p002]

#### Section 2.2: Perspectives of nonparticipants

##### Barriers to attending a school‐based intervention

Nonparticipants outlined why they decided not to enrol in the DISCOVER workshop, with some individuals citing more than one reason. The main reason (*n *=* *8) was that students did not feel able to give up the amount of time that was required. Some (*n *=* *4) reported feeling able to cope with stress by themselves or that the workshop was not necessary for them because they were not particularly stressed. Two students said that they decided not to enrol for the workshop due to their impression of the workshop content.

It was just about missing the lessons, I thought that that was kind of going to add to the stress rather than take it away because just more to juggle with and I just thought at the time it was on I wasn't really ready for missing lessons or anything like that. [NP004]

I would say the time thing was the main reason. […] and then the fact that I wasn't super super stressed then did come into it. It wasn't an urgent priority. [NP005]

I wasn't really 100% sure what the project involved so I didn't really want to commit to something that I wasn't entirely like convinced about at the time. [NP001]

#### Section 2.3: Perspectives of school staff

##### Fit with school values and existing school support

All staff interviewees reported that the workshop was in line with their school values, particularly in terms of student welfare and pastoral care. Three participants commented that the DISCOVER workshop fitted well with the aims of their role (two of these had an exclusively pastoral role in their school).

…rather than having 200 students knocking on my door because they're feeling overwhelmed and need support, I'll only have 100 students. [T002]

All staff valued the DISCOVER workshop at their school, often commenting that it addressed a gap in the support that they were able to provide. Having an external agency come to the school to provide additional mental health support was viewed favourably (*n *=* *5).

…it's quite nice to have people come in, and take some of those students who are really stressed and kind of give them that support that they don't, they can't always get 24/7 with, with us. [T004]

Some staff members (*n *=* *3) highlighted the importance of helping students to become self‐managers of their mental health, and felt that DISCOVER workshops were in keeping with their aims to support students’ personal and emotional development. Some (*n *=* *3) also highlighted the value of the preventative nature of the workshop:

I think the more preventative work we can do the better, really, because I think young people do need to learn to be more resilient and develop skills to develop that resilience, cause you know, life is difficult and there's no getting away from that, but I think we just need to make young people realise that that is normal and how to actually handle it. [T005]

Staff found it difficult to determine the impact of the workshop on students, but one mentioned that the workshop had led to increased peer support. Four staff described the value of normalising mental health problems and creating a culture of openness about stress and/or related psychological difficulties.

So I was walking past two students that were sat in a classroom looking like they were in a very deep and meaningful conversation […] The student was kind of going through the, had been through the programme, was going through, em, techniques on how that they [the other student] could deal with this [personal problem] and trying to help them to problem solve and helping them to feel, to realise that, actually this isn't the end of the world, and let's put this into perspective. Em, could I have seen this student do that with another student before they'd been through the programme? Absolutely not. Because I don't think that student really knew what emotion they were feeling at any particular time or how to deal with that, they were quite kind of angry and frustrated at the time, they're definitely a lot more compassionate and wanting to share their kind of experience… Which I think is really positive thing. [T002]

##### Role in recruitment

School staff (*n *=* *8) played a role in reminding students to attend various aspects of the programme. Most accepted this responsibility, but many (*n *=* *7) felt it was helpful when the DISCOVER team called or sent text messages to prompt students to turn up at the required times. Overall, staff were content with the amount of time required of themselves and their colleagues:

…it will not require that much time and effort but will give a great opportunity to students. [T006]

Because the DISCOVER workshop featured a self‐referral entry route, staff from three of the five schools described putting considerable time and effort into recruitment of particular students to the workshop. They were more comfortable in encouraging groups of students to enrol, with few (*n *=* *2) approaching students individually. Allowing students to opt‐in or self‐refer to the workshop was viewed as important.

…they have to make that decision. That they want to take part in it. I don't think it should be forced upon them, because some students are quite laid back and they don't feel they need it. [T009]

Conversely, two participants said that they had rushed to fill spaces at the last minute. In these cases, recipients of the intervention were not necessarily those who were most in need of support.

##### Staff views on improvements: Clarity regarding workshop remit

Most staff (*n *=* *7) felt they did not receive enough information about the workshop remit and/or expressed a desire to learn more about the specific techniques that were introduced during the workshop. These participants felt that they should have been better informed about the workshop content from the beginning.

Staff (*n *=* *4) were keen to provide follow‐up support after the workshop ended. Some felt they would be better equipped to provide this support if they had received training or resources from the DISCOVER team, which would enable them to remind students of useful stress management techniques after the workshop had ended.

…it would be beneficial for us to be able to have some acknowledgment of what particular strategies work well so that we can reinforce that with students. [T003]

…would quite like to have seen some of the materials that were used […] so that they could kind of continue to use them, or use the right language with them. […] we [staff] don't know quite what happened in those workshops so it, it's difficult to follow‐up… [T007]

##### Overcoming barriers to running the workshop in the future

In response to a specific question about running further DISCOVER workshops, all staff participants said they would want the workshops to return to their school in the future. However, several described some logistical barriers to delivering a workshop programme in the school setting. Most significant of these were timetables and shortage of available classrooms, although none of the logistical issues were described as insurmountable.

## Discussion

This study explored student and school staff perceptions of a school‐based workshop programme (DISCOVER) designed to provide early intervention for adolescents with emotional difficulties. Perceptions of the workshop programme were generally positive, highlighting the acceptability of delivering this type of intervention in the school setting. These findings are promising, as interventions that are viewed favourably by key stakeholders are more likely to be sustainable (Rapee et al., 2006). By allowing key intervention stakeholders to describe their views of the workshop, this study supports an emerging trend to make use of qualitative methods to evaluate complex interventions (Campbell et al., 2000), including programmes focusing on classroom CBT (Boyle et al., 2011).

Schools offer a highly accessible setting for mental health interventions (Masia‐Warner, Nangle, & Hansen, 2006). Students who participated in the DISCOVER workshops described the school as a safe and convenient location. While there were some concerns about confidentiality and fears about making personal disclosures in front of fellow students, students appeared to benefit from being part of a group as it provided opportunities to share and compare experiences with peers. The value of adolescents discussing their problems in a group setting is also noted in a recent study of a Swedish school‐based mental health programme (Garmy et al., 2015).

The UK Department of Health's ‘Future in Mind’ report (Department of Health [DoH], 2015) advocates increased collaboration between the National Health Service (NHS) and UK schools to improve access to mental health support for young people. The present study highlights that some school staff felt collaborations with NHS psychologists could be strengthened by more opportunities to ask questions, share information or learn more about CBT techniques. The importance of clear communication between key stakeholders in delivering interventions has been highlighted by others (Stewart, 2008; Taylor et al., 2014). Both our findings and previous research suggest that stakeholder interactions can be complex, and teachers often want a more active role in developing or delivering workshops (Taylor et al., 2014). Overall, however, school staff who participated in the present study were generally positive about the intervention, perhaps because it provided additional support in contexts of high levels of unmet needs. In fact, many were keen to continue to provide follow‐up support to students after the intervention ended. Given cuts to already stretched child and adolescent mental health services (YoungMinds, 2015), it may be increasingly appropriate for mental health practitioners to upskill and support school staff to respond to common ‘low‐level’ mental health problems within schools, or support students during and following externally delivered interventions.

Students’ preferences for workshop content that is interactive and engaging, preferences for shorter interventions, and school staff requests to be more involved or better informed mirror findings from a process evaluation of a classroom‐based CBT intervention in UK secondary schools (Taylor et al., 2014). The present study adds to the literature by exploring barriers to participation. Reasons for nonparticipation included not having enough time to commit, and being unsure about workshop content. A further contribution is that we explored the views of ‘hard‐to‐reach’ groups. BME groups experience an array of problems in accessing and engaging with suitable psychological services (Lamb et al., 2012). The current findings add to this literature by indicating that BME young people may struggle to engage with interventions even after overcoming barriers associated with accessing them. In comparison to White British workshop participants and those who had previously sought help for emotional difficulties, BME students who had no previous experience of accessing mental health support had particular difficulty with the goal‐setting component of the workshop programme, and were more likely to voice concerns about their ability to achieve their goal. Perceptions of self‐competence may vary between different cultural groups (Schmitt & Allik, 2005) and it is possible that past experience of help‐seeking could increase confidence in working towards a goal. This suggests that clinicians delivering interventions with a goal‐setting component should be particularly attentive to the needs of BME students, and aware that those who have not previously sought help may require additional guidance and encouragement. In particular, individuals from hard‐to‐reach groups may not understand their problems in the same way as service providers, leading to difficulties in communicating the problems that they are facing (Lamb et al., 2012). This may impact on students’ ability to talk about the issues that they face and recognise which techniques and goals might help them to overcome these.

### Research and clinical implications

This study supports the use of qualitative methods to explore aspects of school‐based interventions that could not have been investigated using quantitative outcome measures alone. In particular, the qualitative methodology provided insights into nuances of the acceptability of group‐based mental health support for older adolescents.

Findings suggest that delivery of similar interventions may benefit from ensuring that stakeholders (school staff and students) fully understand the nature of the intervention and the value of participation. This may have important implications for recruitment, acceptability and the effectiveness of such interventions. Key challenges raised by school staff relate to logistical issues (e.g. room bookings; scheduling). A longer planning phase, where the intervention team liaises closely with a designated member of school administrative staff, may be beneficial. The findings of this study suggest that clinical psychologists (and other specialist external providers) involved in school‐based interventions may need to rethink the nature of their partnerships with school staff, with greater focus on sharing expertise and building capacity among staff with pastoral roles who have an appetite and will to learn from them. A further challenge is that staff were comfortable in encouraging groups of young people to participate, but less willing to approach students on a one‐to‐one basis.

The struggle to address the mental health needs of adolescents is widely acknowledged by parents, educators, psychologists and health professionals. The DISCOVER workshop programme was designed to offer a way to address this need in UK schools. The present study suggests that a group‐based intervention, delivered in a familiar setting, with content that is engaging, interactive and personalised, may offer an appealing alternative to the traditional one‐to‐one therapeutic relationship for this age group. Wider dissemination of this, or similar interventions, would not only reduce demand on traditional NHS mental health services but also would potentially normalise help‐seeking for mental health issues in the school setting, thus helping young people to reach out when they need support.

### Strengths and limitations

Our study triangulated the perspectives of three stakeholder groups (participating and nonparticipating students, and school staff), enabling a rounded picture of intervention acceptability to be obtained. The inclusion of nonparticipants allowed us to capture key barriers to participation that can be considered in future iterations of this and similar programmes. However, despite intensive recruitment efforts, in the case of the nonparticipant sample, there were some schools where no young people agreed to be interviewed. More female than male students participated in this study, reflecting the gender ratio of those attending the workshops overall. The findings therefore reveal more about females’ experiences of accessing services for emotional difficulties. Further efforts are required to develop mental health interventions for males, and to help address gender‐specific access issues. Although data were collected by a researcher who was not involved in delivery of the workshop, for some study participants, knowing that she was affiliated with workshop psychologists may have impeded open expression of their views. Due to the duration of time between the workshop day and the collection of interview data given constraints posed by the RCT follow‐up, aspects of student and staff accounts may have been misremembered or inaccurate.

## Conclusion

This paper outlines some of the successes and limitations of delivering a self‐referral school‐based mental health intervention to older adolescents. Qualitative evaluation of the DISCOVER programme indicated that running a workshop with telephone follow‐up is feasible in Inner London schools, accessible to students, including those from BME groups, and was considered acceptable by participating students and involved school staff. This study has various practical and clinical implications, including the value of designing and delivering school‐based interventions that are interactive and personalised. Such findings can help to inform future iterations of the DISCOVER programme, and provide useful insights for others looking to develop or strengthen similar interventions.

## Ethical information

Ethical approval for the DISCOVER Project was granted by the Health Research Authority NREC Committee London ‐ Camberwell St Giles: ref 14/LO/1416. All participants provided written informed consent and consent for their interview to be audio‐recorded.
